# Teacher Motivation and Burnout of English-as-a-Foreign-Language Teachers: Do Demotivators Really Demotivate Them?

**DOI:** 10.3389/fpsyg.2022.891452

**Published:** 2022-04-27

**Authors:** Masatoshi Sato, Francisca Fernández Castillo, Juan Carlos Oyanedel

**Affiliations:** ^1^Department of English, Universidad Andres Bello, Santiago, Chile; ^2^Trewhela’s School, Santiago, Chile; ^3^Faculty of Education and Social Sciences, Universidad Andres Bello, Santiago, Chile

**Keywords:** teacher motivation, demotivators, burnout, second language motivation, self-determination theory, non-native-speaking teachers, COVID-19, mixed methods research

## Abstract

This study examined the relationships between teacher motivation (TM) and perceived burnout of English-as-a-foreign-language (EFL) teachers in Chile. A particular focus was given to demotivators and their impact on TM and burnout. The impact of COVID-19 was considered. Given that EFL teachers tend to be second language (L2) learners of English themselves, the study also investigated how TM and L2 motivation interact with each other. The participants were 154 school-level teachers with a range of backgrounds (teaching experience, geographic areas, and school sectors). In the questionnaire, four scales were included: (a) autonomous motivation for teaching; (b) demotivators; (c) perceived burnout; and (d) L2 motivation. Fifteen teachers were interviewed in order to triangulate the survey results. Structural equation modeling showed that TM negatively predicted perceived burnout, suggesting that it can counter teachers’ emotional exhaustion and their perceived lack of personal accomplishment. Demotivators predicted TM positively, albeit weakly. L2 motivation was found to be only weakly related to TM. Qualitative findings indicated that teaching experience mediated the role that demotivators played in relation to TM. Experienced teachers, especially those who held intrinsic motivation to teach, channeled the impact of demotivators, including those relating to the pandemic, to a positive motivational force to teach. The study implies the importance of considering teachers as agents and devising an educational system in which their mental health is prioritized.

## Introduction

Without teachers, there is no teaching. The attrition rate of teachers is high globally (OECD, 2021) and the teacher shortage has been an issue for decades (Toropova et al., 2021). The attrition rate of second language (L2) teachers is also high (Swanson and Mason, 2018), and Chile—the context of the current study—is no exception. The survey conducted by Universidad de Chile analyzed the attrition rate of pre-service and in-service teachers across the country between 2005 and 2016 (Centro de Investigación Avanzada en Educatión, 2018). Results showed that between 6 and 12% of teachers left the profession in the first year. After 10 years of working, around 30% had left their teaching jobs. When there is an insufficient number of qualified teachers at a school, the quality of education naturally decreases and student learning is negatively affected (Young, 2018). To this end, research has shown that a primary cause of leaving the teaching profession is burnout (Madigan and Kim, 2021), and that burnout is significantly affected by teacher motivation (TM: Alexander et al., 2020). When a teacher does not feel motivated to teach, they feel emotionally exhausted and professionally unaccomplished. As a consequence, they may quit the job. Even when a teacher does not ultimately quit, the quality of education suffers when they are not motivated to teach (Bardach and Klassen, 2021). The current study, therefore, explored the relationship between TM and perceived burnout.

While there are a number of studies showing the connection between TM and burnout (see Slemp et al., 2020), no similar research exists in the context of the current investigation. Also, previous research has largely focused on factors that motivate teachers to teach. While boosting the impact of motivators is one way of motivating teachers, another way is to decrease the impact of factors that demotivate them. However, there is little research as to (a) what factors demotivate teachers, (b) how demotivators interact with TM, and (c) whether demotivators influence perceived burnout. With the pandemic (COVID-19) unexpectedly forcing teachers to teach online without much training or sufficient resources (Beardsley et al., 2021), investigation into TM and demotivators is especially crucial. By focusing on English-as-a-foreign-language (EFL) teachers, the current study examined the impact of demotivators on TM as well as perceived burnout.

Meanwhile, research has shown that the focused constructs—TM, demotivators, and burnout—are affected by context, including the subject that they teach (mathematics, music, special education, etc.). Despite the recent increase in teacher psychology research in the L2 field (see Dörnyei and Ushioda, 2013; Kubanyiova and Feryok, 2015; Mercer and Kostoulas, 2018; Gkonou et al., 2020), TM remains a somewhat under-investigated construct (Dörnyei and Kubanyiova, 2014; Lamb and Wyatt, 2019). Given the impact that TM may carry on educational quality in other disciplines as well as discipline-specific factors potentially influencing TM, it is pivotal to examine (a) whether TM of L2 teachers exhibits a similar tendency and (b) factors mediating the impact of TM held by L2 teachers. Context also includes where teachers are situated. This is partly because each teaching context comes with unique educational policies and societal views of the teaching profession (Collie et al., 2016). The impact of context-specific variables may be enhanced for EFL teachers. Unique to EFL education is that teachers tend to be learners of the language. It is reasonable to hypothesize that EFL teachers’ motivation may be related to motivation to learn the language (see Rahmati et al., 2019). That is, some teachers may like to learn the language and, hence, decide to become or continue to be an L2 teacher. Theoretically, the vast majority of both TM and L2 motivation research is based on self-concepts, such as self-determination theory (SDT: Deci and Ryan, 1985). In this framework, what drives the action forward, either learning an L2 or teaching an L2, is explained by the ways in which people view themselves (actual self) and what they aspire to be in the future (future self). However, empirical research into TM and L2 motivation is rare. The current study included L2 motivation as a potential antecedent of the TM of EFL teachers.

## Literature Review

### Teacher Motivation and Burnout

SDT is arguably the most frequently used theoretical framework in motivation research including TM (see Urdan et al., 2014; Richardson and Watt, 2018). The theory provides a broad framework for understanding how people are intrinsically and extrinsically motivated, or not motivated, to take an action. It focuses “on people’s inherent motivational propensities for learning and growing, and how they can be supported” (Ryan and Deci, 2020, p. 1). Roth et al. (2007) applied SDT to TM and developed a questionnaire. Results with 132 Israeli school teachers confirmed the theoretical structure of TM, whereby the construct consists of a continuum from more autonomous to more controlled. While high TM predicted a sense of personal accomplishment, low TM predicted emotional exhaustion. TM is also related to many other constructs. Slemp et al.’s (2020) meta-analysis (*N* = 102 with 1,117 correlation coefficients) revealed that when teachers are autonomously motivated (that is, with intrinsic and integrated motivation), their general well-being and job satisfaction tend to be higher. However, when their TM is governed by controlled regulation (with introjected and external motivation), their stress levels tend to be higher.

Slemp et al.’s (2020) study also found TM to be negatively correlated with perceived burnout. Originally coined by Maslach and Jackson (1981), burnouts are characterized by three dimensions: (a) emotional exhaustion, involving feelings of helplessness, hopelessness, and entrapment; (b) depersonalization, pertaining to a physical exhaustion caused by low energy and chronic fatigue; and (c) reduced personal accomplishment, based on the development of negative reactions to the work, people, and self. A number of studies have revealed more correlates of burnout. For instance, with Norwegian high school teachers (*N* = 262), Skaalvik and Skaalvik (2020) showed that when teachers were emotionally exhausted and felt they were not satisfied with their job performance, they tended to think about leaving the profession. However, their job satisfaction negatively predicted motivation to quit the job. It would be interesting to see whether TM functions in a similar way, deterring perceived burnout of EFL teachers.

### Factors Affecting Second Language Teacher Motivation

Although research has shown that the teaching profession is generally considered a stressful one (Kim et al., 2019), specific factors that motivate or demotivate teachers have been relatively under-investigated. Knowing those factors would take us beyond understanding TM as a psychological construct and may lead to ideas for interventions designed to increase TM. In reviewing specific TM factors, we operationally define factors that positively affect TM as *motivators* and those that affect TM negatively as *demotivators*. Some factors may influence TM depending on their degrees (*mediators*) (see Schiefele, 2017). For instance, when a teacher is given financial resources, this could positively affect TM. When another teacher lacks financial resources, however, the same factor may negatively influence TM. In order to arrive at deterrence strategies for decreasing TM, we focused on demotivators and mediators specifically in relation to L2 teachers.

A number of situational- and individual-specific factors have been found to demotivate L2 teachers. Some prominent demotivators include a variety of school duties and heavy workload (Kumazawa, 2013), limited financial resources (Yaghoubinejad et al., 2017), unexpected classroom realities coupled with students’ expectations (Tao et al., 2019), students’ classroom attitudes (Sugino, 2010), government policies (Song and Kim, 2016), and geographical location (Gao and Xu, 2014), to name a few. In addition to those external factors, TM may be eroded when wider society does not appreciate the teaching job and views the profession as somewhat socially inferior (Alexander et al., 2020), a perception sometimes fueled by the media (Hettiarachchi, 2013). TM can also be influenced by specific people with whom teachers have interacted. For instance, some pre-service teachers may feel demotivated by their instructors when they receive comments that discourage them from becoming a teacher (Farrell, 2015). Unique to the current educational environment is the pandemic, which has been shown to have led to a significant stressor for L2 teachers. Appel and Robbins (2022) surveyed L2 teachers of various languages in Spain in March 2020 (*N* = 64). Results showed that only 2% reported no effects of COVID-19 on their emotions. Although it is unquestionable that COVID-19 has negatively affected teachers’ well-being, we still do not know how the pandemic has impacted on TM.

Those potential demotivators may accelerate the discrepancy that a teacher perceives between what they are now (their actual self) and the person that they aspire to be (their ideal self). Identifying the discrepancy, however, does not necessarily lead to reduced motivation. As Gao and Xu (2014) put it, self-discrepancies are “likely to become the driving forces in their professional development and career pursuit” (p. 153). In other words, it is theoretically possible that demotivators drive teachers forward to take an action to further pursue their careers (called internalization). The same can be said for the impact of COVID-19. It is possible that teachers with high intrinsic TM may be less susceptible to the forced changes to the online teaching format because their motivation is based on internal forces for improving their practice. The current study empirically tested this possibility.

Teaching experience has arguably been found to have a notable mediating effect on TM. As teachers gain experience, they may learn how to cope with demotivators and solidify their professional identities, which in turn influences TM (Csizér, 2020b). In the Chilean context, Díaz Sacco et al. (2021) conducted a longitudinal study following teachers from 2007 to 2018 (*N* = 2,701,574). They confirmed that the attrition rate was much higher in the first few years of the teaching career, gradually decreasing as teachers gained experience. Different from teaching experience is the comparison between pre-service and in-service teachers. While in-service teachers’ motivation is affected by factors that they have some experience of, pre-service teachers’ motivation is largely based on their predictions of the reality of the teaching job (Bergmark et al., 2018). With pre-service EFL teachers in China, Yuan and Zhang (2017) found that teacher education programs, including practicum experiences, significantly impacted on pre-service teachers’ motivation and on whether they finally decided to become L2 teachers. In a similar context, Zhang et al.’s (2020) survey with 442 pre-service teachers showed that their TM was largely intrinsic in nature, perhaps because they held idealistic beliefs about the job prior to entering the occupation. However, Wang and Zhang’s (2021) interview study showed that pre-service TM was driven by external factors, such as income.

In the present study, we collected data from both pre-service and in-service teachers with a range of teaching experience in different contexts (rural vs. urban; private vs. public). This way, we endeavored to control the impact of those factors on the overall relationships among TM, demotivators, and perceived burnout.

### Second Language Motivation and Teacher Motivation

The most common theoretical framework for the current L2 motivation is L2 motivational self-system (L2MSS), developed by Dörnyei (2005). Like TM and burnout, L2MSS is based on the self-concept (Csizér, 2020a). While *ideal L2 self* is based on L2 learners’ future images (i.e., vision) incurring aspirations and hopes, *ought-to L2 self* pertains to attributes that learners believe that they should possess. Those internal and external motivational forces have been shown to predict L2 learning outcomes by many studies (see Al-Hoorie, 2018; Sato, 2021). Though there is a limited number of studies examining the relationship between L2 motivation and TM, Rahmati et al. (2019) applied L2MSS to TM held by Iranian EFL teachers. The newly developed questionnaire, focusing on TM and vision (how teachers envisioned themselves in the future), functioned moderately well (*r* = 0.59), showing that teachers who had clearer future vision tended to be more motivated to teach. However, Kubanyiova (2006) characterized L2 motivation as a “wrong” type of TM (p. 6). This was because affiliation a teacher feels toward the language they teach does not necessarily relate to intrinsic motivation to teach. It is likely that L2 learning experience (cf. L2 learning motivation) may impact on TM and teachers’ approach to their students (Csizér, 2019). However, there is scant research investigating the relationship between L2 motivation and TM.

Based on the above literature, the following research questions were explored:

RQ1: How does teacher motivation of Chilean EFL teachers relate to their perceived burnout?

RQ2: What roles do demotivators play in relation to teacher motivation and perceived burnout?

RQ3: Is L2 motivation related to motivation to teach the L2?

## Materials and Methods

### Context

Because TM is inevitably affected by context and encompasses the sociocultural status of the target language, educational system, and government policies, we first detail the context of the current study. As the utility of English for social and professional opportunities continues to grow, the Chilean government puts emphasis on improving English education (Barahona, 2015). In this context, as with the case in other EFL contexts in the world, the majority of English teachers are those who learned English themselves (Sato and Oyanedel, 2019). In fact, data from the current study showed that only 0.02% of respondents were L1-English speakers. Consequently, the responsibilities of teacher education programs include not only training of pre-service teachers but also teaching of English itself (Vivanco Torres, 2016). Teacher training programs at universities are required to follow the government guidelines entailing specific classes to be taught and practicum requirements with different age groups (Martin, 2016).

Meanwhile, English is a compulsory subject from Grade 5 to Grade 12 in Chile (Currículum Nacional, 2020). The general proficiency level of Chilean English learners struggles to meet the desired goal, however. Education First (EF: 2021) ranked Chile as 42^nd^ among 112 countries in terms of general English proficiency levels. One of the mediating factors for more successful learning outcomes is the educational sector. The government reported that while 85% of students with higher socioeconomic statuses reached the basic or intermediate proficiency levels, only 9% with lower socioeconomic statuses did so (Agencia de Calidad de la Educación, 2019). This implies differences in teachers’ working environments, the resources that they are given (time and money), and the students they teach (English proficiency levels as well as general attitudes), depending on the school they work at (public, private, or subsidized). The current study considered these contextual variables.

### Participants

A survey invitation was sent out to English teachers throughout the country in 13 regions, with the help of local teacher organizations as well as teacher training programs of four major universities in Santiago areas. We contacted the universities in order to recruit pre-service teachers. The four universities (two public and two private) shared similar curricula including required courses of applied linguistics, second language acquisition, and practicum courses at local schools. Of these pre-service and in-service teachers, 155 completed the survey. They lived in 10 different regions. One teacher whose first language was English was excluded from the dataset because the current study examined the relationship between TM and L2 motivation. The final dataset therefore consisted of 154 responses.

Respondents included 96 female and 33 male teachers (25 preferred not to say). Their ages ranged from 18 to 56, with an average age of 29.84 years (*SD* = 9.45). Nearly two-thirds (65%) were full-time teachers. They worked at either public (21.6%), private (27.5%), or subsidized schools (20.6%), or other types of language institutions (2.9%). The rest described themselves as independent language teachers (27.4%). The majority of teachers (69.6%) reported that they had completed a teacher training program and 22% were pre-service teachers. In-service teachers’ experience varied from 1 to 35 years of teaching (*M* = 8.01; *SD* = 8.12).

In order to gain a fuller understanding of TM, in-depth interviews were conducted. Teachers were contacted when they had expressed an interest in the questionnaire in participating in interviews. From those, we purposefully selected 15 teachers to include variability in the factors that could have affected the results, such as teaching experience and educational sector. As a result, the interviewees included four pre-service and 11 in-service teachers whose ages ranged from 20 to 40 (2 teachers preferred not to disclose their age). There were 12 female and three male teachers. All spoke Spanish as their first language and learned English as their L2. The in-service teachers’ experience ranged from 1 to 34 years. Two worked at public schools, six at private schools, and three at subsidized schools.

### Data Collection

Research has shown that factors specific to local contexts mediate TM, yet there is little TM research in the Chilean context. Hence, a mixed-methods design was used in seeking “the most informative, complete, balanced, and useful research results” (Johnson et al., 2007, p. 129). This design answers a call for a nuanced understanding of teacher psychology in general (Kubanyiova and Feryok, 2015), as well as methodological recommendations to contextualize L2-related issues in instructed settings (Sato, 2022). In particular, a large-scale survey was combined with in-depth interviews to triangulate the results. The data were collected in September to November 2020—in the midst of the pandemic.

#### Questionnaire

Rather than assuming that L2 teachers’ motivation is associated with their L2 motivation, the current study used instruments that have been validated in general educational research. This way, we hoped to tease apart TM as a distinct construct from L2 motivation. Three validated scales were adopted (TM, perceived burnout, and L2 motivation) and one scale was newly developed (demotivator questionnaire) due to the lack of relevant instruments. Importantly, the first three scales were all based on self-concepts so as to ensure theoretical congruency in the structural model (see the “Data Analysis” section). One item was added to consider the impact of COVID-19 on TM (“The situation caused by the current pandemic affected my motivation to teach negatively.”). Participants were asked to respond using a six-point Likert scale (1 = strongly disagree; 6 = strongly agree). The questionnaire ended with a section focusing on demographic variables, the results of which are reported in the Participants section. The complete questionnaire can be found in Supplementary Material.

##### Autonomous Motivation for Teaching

TM was examined with Roth et al.’s (2007) battery for autonomous motivation for teaching. The questionnaire was developed based on SDT and designed to capture “teachers’ thoughts and feelings regarding their own motivations for engaging in teaching” (Roth et al., 2007, p. 761). It consists of 16 items in total with four subconstructs: *external motivation*, *introjected motivation*, *identified motivation*, and *intrinsic motivation*. The subconstructs are placed on the spectrum of perceived autonomy, the most autonomous being intrinsic motivation (e.g., “When I invest effort in my work as a teacher, I do so because I enjoy finding unique solutions for various students.”), and with the least autonomous as external motivation (e.g., “When I try to find interesting subjects and new ways of teaching, I do so because I want the parents to be satisfied so they won’t complain.”). The scale has been validated by numerous studies and found to be related to other constructs such as teacher well-being (e.g., Reeve and Cheon, 2021) and students’ learning outcomes (e.g., Bardach and Klassen, 2021). The data from the current study yielded an overall internal consistency score of α = 0.79, with the four subconstructs: external motivation (α = 0.80); introjected motivation (α = 0.75); identified motivation (α = 0.70); intrinsic motivation (α = 0.70).

##### Teacher Demotivators

A teacher demotivators questionnaire was developed. This was primarily based on Sugino’s (2010) study, for several reasons. First, Sugino’s study focused on the TM of EFL teachers. Second, the study’s context was deemed to share some key factors with the current study. These include L2 teachers having learned the L2 themselves, and the socio-educational status of the target language. Both in Japan and Chile, students do not have much chance to be exposed to English, yet the traditional grammar-translation methods prevail as a common teaching method. Consequently, general English proficiency levels struggle to advance at national levels, potentially influencing teachers’ own English proficiency as well as their motivation to teach. Also, in both contexts, English is generally considered a ticket to social success.

In Sugino’s (2010) study, 37 demotivators were tested with 97 EFL teachers. Results yielded seven demotivators as prominent and internally consistent. The current study used those seven items (e.g., students’ attitudes, focus on tests, and high workload) and added three deemed to be relevant to the current study’s context (e.g., public policy and low pay). The added items were based on previous L2 research focusing on the Chilean context. The internal consistency of the 10-item teacher demotivator questionnaire resulted in α = 0.79.

##### Perceived Burnout

Perceived burnout was measured with Friedman and Farber’s (1992) teacher burnout scale. Based on the Maslach Burnout Inventory (MBI: Maslach and Jackson, 1986), Friedman and Farber developed a 15-item burnout scale. As there is limited research on burnout of L2 teachers, the scale was deemed appropriate because of its wide use with teachers of other content areas (see Saloviita and Pakarinen, 2021). The Cronbach’s alpha of the scale reached 0.76.

##### Second Language Motivation

L2 motivation was measured with the scale developed under L2MSS (Dörnyei, 2009). This scale was also developed based on the self-framework and contains two types of self: *ideal L2 self* and *ought-to L2 self*. Ideal L2 self-concerns ways in which L2 learners see themselves as L2 users in the future (e.g., “I can imagine myself living abroad and using English effectively for communicating with the locals.”). Ought-to L2 self-entails attributes that L2 learners believed they should possess based on the views of others, such as parents and society (e.g., “Studying English is important to me in order to gain the approval of my peers/teachers/family/boss.”). The items were taken from Dörnyei and Taguchi (2010) and the scale consisted of 15 items in total. The overall reliability score of the scale reached 0.81 (ideal L2 self: α = 0.91; ought-to L2 self: α = 0.85).

#### Interviews

Semi-structured, in-depth interviews were based on 10 prompts deriving from Rahmati et al. (2019), who investigated the motivation and vision of Iranian EFL teachers. The prompts included topics such as: the initial motivation to become a teacher; factors that motivated or demotivated them to teach; factors that may motivate them in the future; and the impact of teaching experience. Several questions specific to pre-service teachers were added (e.g., “Do you think your environment (parents, friends, and society) affects your motivation to become a teacher?”). The complete list of the prompts is included in Supplementary Material. Interviews were conducted in the teachers’ L1 (Spanish), individually *via* an online platform. Each interview took between 30 and 45 min, totaling 490 min. All interviews were audio-recorded.

### Data Analysis

#### Questionnaire

In exploring the relationships among TM, teacher demotivators, and perceived burnout, structural equation modeling (SEM) was used (RQ1 and RQ2). The targeted constructs were set as latent variables. Drawing on the previous research, the models were fitted using AMOS 24.0. The first procedure involved the imputation of missing data for those participants who skipped some items (Mode of missing items = 2; percentage of cases with missing data: 12%). Second, the data were analyzed and imputed using the multiple imputation procedure of IBM SPSS 24. For all scales, linear factors were estimated using exploratory factor analysis, and Bartlett factor scores were calculated and saved for use in SEM.

To answer RQ3, bivariate correlations (Pearson, two-tailed) were explored among the subscales of TM and L2 motivation. With no empirical evidence or theoretical framework that can be used to hypothesize a structural model, the relationship between L2 motivation and TM was examined separately from the SEM. Instead, an exploratory, associative design was chosen.

#### Interviews

As research of Chilean EFL teachers’ motivation was scant, and TM was thought to be context specific, we drew on some of the principles of grounded theory (Corbin and Strauss, 2015) in exploring the dataset. First, we explored the transcripts to look for recurring themes which led to three overall categories: *motivators*, *demotivators*, and *mediators*. Specific themes emerging from the initial analysis were explored again in the transcripts, in order to increase the validity of the coding. Finally, interrater reliability was obtained by having another researcher code 20% of the dataset, which yielded κ = 0.98. Disagreements were resolved by discussion between the first author and the initial coder. The results were used to answer all three RQs.

## Results

### Questionnaire

**FIGURE 1:**
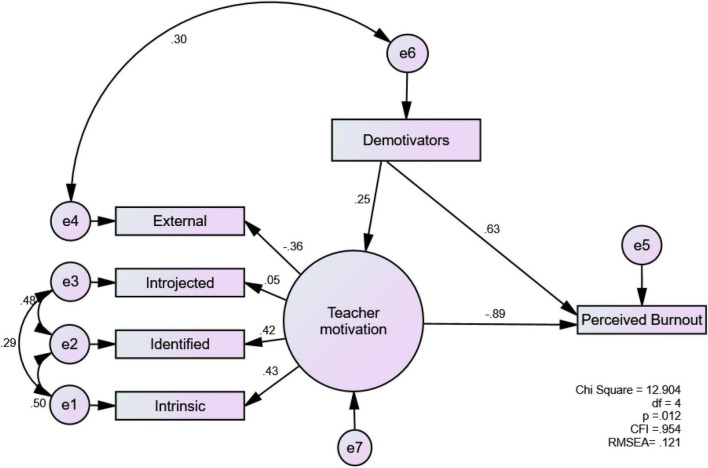
Standardized model of teacher motivation, teacher demotivators, and perceived burnout (*N* = 154).

Table 1 includes the descriptive statistics of the focused variables. RQ1 and RQ2 asked whether and how TM, demotivators, and perceived burnout were related to each other. Figure 1 shows the structural model. The goodness of fit indices show the data fit the model well (χ^2^ = 12.904; CFI = 0.954). The RMSEA score (0.121) suggests that there are possible unexplained correlated errors in the model; however, the narrow 90% CI [0.051–0.197] suggests a moderate fit (see Zhang et al., 2021). In addition, the TM scale was shown to reflect its theoretical framework: as the autonomy of teaching moves from external to intrinsic, the coefficients increased: –0.36 < 0.05 < 0.42 < 0.43.

**TABLE 1:** Descriptive statistics of the focused variables (*N* = 154).

Variables	*M*	*SD*	Range
TM-external	2.88	1.16	1.00–5.50
TM-introjected	4.34	1.11	1.00–6.00
TM-identified	5.49	0.73	1.00–6.00
TM-intrinsic	5.18	0.84	1.00–6.00
COVID-19	3.28	1.53	1.00–6.00
Demotivators	4.07	0.84	1.00–6.00
Perceived burnout	3.27	0.65	1.00–5.27
L2M-ideal self	5.30	0.76	1.00–6.00
L2M-ought to self	2.23	0.91	1.00–5.25

*TM, teacher motivation; L2M, L2 motivation.*

In terms of the relationships, the three hypothesized paths were significant: TM→burnout = –0.89; demotivators→burnout = 0.63; demotivators→TM = 0.25. First, the TM’s predicting power of burnout was strong (–0.89). This result shows that teachers with higher TM tended to perceive less burnout. Conversely, demotivators positively predicted perceived burnout (0.63), indicating that teachers who felt demotivated by the focused factors tended to feel more burnout. Finally, demotivators predicted TM positively, albeit with a small effect (0.25). Given (a) the relationship between external TM and demotivators (0.30), and (b) the negative predicting power of external TM in relation to the latent variable of TM, demotivators were thought to be primarily associated with external TM.

**FIGURE 2:**
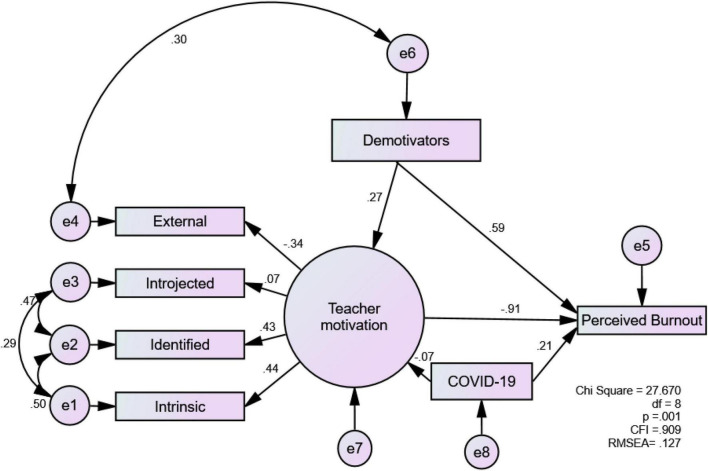
Standardized model of teacher motivation, teacher demotivators, perceived burnout, and impact of COVID-19 (*N* = 154).

The second model considered the impact of COVID-19. The general structure did not change very much (Figure 2). The goodness of fit indices were acceptable (χ^2^ = 27.670; CFI = 0.909), although the RMSEA score (0.127: 90% CI [0.077–0.180]) was above the suggested level. Notably, COVID-19 did not impact on TM very much (–0.07), yet it did predict perceived burnout (0.21). This pattern is evident when the path coefficients between TM and burnout with (–0.91) vs. without (–0.89) COVID-19 in the two models were compared. The difference indicates that although COVID-19 did not impact on TM very much, it indirectly predicted burnout *via* TM.

RQ3 explored the relationship between L2 motivation and TM. Table 2 displays the correlation coefficients and *p*-values. The relevant results are highlighted (the internal structure of TM was reported *via* SEM). First, the correlation coefficients were all low (ranging from 0.037 to 0.295). Second, clear gradual changes in the degrees of associations were found. Ideal L2 self is associated with external TM the least (*r* = 0.037), and the degrees of association gradually increase toward intrinsic TM (*r* = 0.295). Ought-to L2 self exhibits an opposite pattern, whereby it is associated the most with external TM (*r* = 0.290) and the least with intrinsic TM (*r* = 0.012).

**TABLE 2:** Correlations between teacher motivation and L2 motivation (*N* = 154).

	TM-external	TM-introjected	TM-identified	TM-intrinsic	L2M-ideal self	L2M-ought to self
**TM-external**	—					
TM-introjected	**0.248** **(0.006)**	—				
TM-identified	0.033 (0.722)	**0.514** **(0.000)**	—			
TM-intrinsic	–0.099 (0.280)	**0.342** **(0.000)**	**0.585** **(0.000)**	**—**		
L2M-ideal self	0.037 (0.691)	**0.191** **(0.036)**	**0.280** **(0.002)**	**0.295** **(0.001)**	—	
L2M-ought to self	**0.290** **(0.001)**	**0.188** **(0.039)**	0.113 (0.217)	0.012 (0.900)	0.062 (0.501)	—

The figures in the parentheses show the p-values. TM = teacher motivation; L2M = L2 motivation. Relevant correlations are highlighted. Significant correlations are marked in bold. *p < 0.05.

### Interviews

The analysis of interview data resulted in 237 coded quotes with three overall categories—*motivators*, *demotivators*, and *mediators*—entailing multiple themes within. Table 3 reports the frequencies of each theme. As the nature of most of the themes is self-evident, we will highlight some prominent ones here. We will use additional interview quotes to triangulate the questionnaire results in the “Discussion” section.

**TABLE 3:** Results of qualitative analysis of interview data.

Category	Theme	*f*	% (within category)
Motivators (*f* = 55)	Passion for teaching	21	38.2
	Teaching as a skill	17	30.9
	English as a language	10	18.2
	Student learning	7	12.7
Demotivators (*f* = 67)	Public policy	24	35.8
	Lack of appreciation of teaching profession	13	19.4
	School policy	7	10.4
	Curriculum restrictions	7	10.4
	Focus on tests	5	7.5
	Large class size	5	7.5
	Long teaching hours	3	4.5
	Students’ misbehaviors	3	4.5
Mediators (*f* = 114)	Collegiality among colleagues	31	27.2
	Financial resources	21	18.4
	Evaluation from others	16	14.0
	School support	14	12.3
	Influence from others	11	9.7
	Salary	5	4.4
	Teacher training program	5	4.4
	Students’ English proficiency	4	3.5
	Students’ motivation	4	3.5
	Time	3	2.6

*Themes are rank-ordered based on their frequencies.*

*Motivators* were defined as factors that drive pre-service and in-service teachers forward to choose or to continue within the teaching profession (*f* = 55). Among those, *passion for teaching*—personal passion for the act of teaching itself—was mentioned the most (*f* = 21). For instance, Rosa (34 years of teaching experience; subsidized school) said: “I left my teaching job for a year but then I understood that my vocation is teaching. It is what I like and I am going to die being a teacher”. The second most mentioned theme was *teaching as a skill* (*f* = 17) that entailed a pursuit of skills related to teaching. Eleven teachers mentioned that the pandemic forced them to learn new teaching skills, especially those related to technology use. For instance, Fernanda (17 years of teaching experience; private school) said: “We are living in times of pandemic. It has been a difficult time, especially the online classes, but I have felt motivated to seek interactive activities and games. I am mastering the computer, the internet, and different apps.” Interestingly, factors that have been reported to motivate teachers in previous studies were not found very much in the current dataset: namely, *English as a language* (*f* = 10) and *student learning* (*f* = 7).

*Demotivators* included eight themes that discouraged teachers from choosing or continuing the teaching profession (*f* = 67). The strongest demotivator was found to be *public policy* (*f* = 24), which included not only English education policies themselves but policy-making processes. The majority of teachers shared their frustration with the fact that politicians often do not have a teaching background. Franco (13 years of teaching experience; private school) stated that “there are many people behind the system who have nothing to do with education, who are not people who are in the classroom all day knowing the reality.” Another oft-mentioned theme was *appreciation of teaching profession* (*f* = 24) which pertained to societal judgment of the teaching job. For example, Lorena (pre-service) commented that “people always say “education is super important” but “the teachers” are never in the discussion.” Other demotivators commonly identified in previous research (e.g., class size, teaching hours, and students’ behaviors) did not appear in the current study very much.

*Mediators* included factors that affected the degree of TM either positively or negatively. This category was found most frequently in the dataset (*f* = 114) and the prominent themes pertained to both internal and external motivational factors. The most frequently mentioned theme was *collegiality among colleagues* (*f* = 31). Colleagues in this sense included other teachers, administrators, parents, and students. In essence, teachers observed that when they perceived their working environment to be supportive and collaborative, they felt motivated to teach. While Rosa said “I feel that my colleagues are like friends and families,” Javiera (27 years of teaching experience; subsidized school) explained: “What may improve my motivation, which unfortunately I don’t have right now, is a team that supports, understands, listens, and collaborates with us.” There were some themes that emerged from the pre-service and early career teachers in particular. *Influence from others* (*f* = 11) affected teachers’ initial motivation to take up the role. Renata (1 year of teaching experience; private school) reflected that “When I started my practicum, the teacher from the first school I went to was so awesome that she motivated me and made me want to actually be a teacher.” *Teacher training program* (*f* = 5) was mentioned by five teachers, including Isidora (3 year of teaching experience; private school) who said: “Unfortunately, the university in which I studied gave tools for prestigious and privileged schools, but not for the reality of the majority of schools in Chile.” Yet, Francisco (3 year of teaching experience; public school) said: “I think it was during my university studies when I actually started to get motivated with the profession.”

## Discussion

### Second Language Teacher (De)motivation and Burnout

RQ1 explored the relationship between TM and perceived burnout of Chilean EFL teachers. First, results support the recent aggregated findings related to the internal structure of TM (e.g., Slemp et al., 2020). While external and introjected motivation (together as controlled motivation) negatively contributed to the latent construct of TM, integrated and intrinsic motivation (together as autonomous motivation) positively contributed to TM. Furthermore, TM negatively predicted perceived burnout, showing that it can be an effective deterrent to burnout. Given the differential contributions of the four subconstructs of TM, it appears that burnout is negatively correlated with teachers’ internal views of their profession, rather than external factors such as their working environments and curricular restrictions (see Friedman and Farber, 1992). In other words, when teachers are internally motivated, they tend to perceive less burnout, highlighting the importance of intrinsic motivation for preventing teachers from quitting the job. The interview results support such an interpretation. When the themes from the interviews are categorized as either intrinsic or external, the top two motivators (i.e., *passion for teaching* and *teaching as a skill*) are internal in their nature, while the top two demotivators (i.e., *public policy* and *lack of appreciation of teaching profession*) illustrate how external factors influence TM negatively.

The current study focused on demotivators and mediators. A surprising finding was that the external factors that demotivated teachers in other contexts (i.e., class size, teaching hours, and students’ behaviors) did not appear to be strong demotivators for the Chilean EFL teachers. On the one hand, the study supports the idea that TM is highly context specific. On the other hand, it is possible that the timing of the data collection—during the pandemic—affected the results. Due to the forced shift to online teaching, teachers did not face “typical” teaching issues, such as classroom management with a large number of students. The testing formats had also changed. Consequently, factors that teachers perceived to be negative shifted toward value-related issues (e.g., public policy and societal appreciation; see Appel and Robbins, 2022). As explained, teachers’ frustration related to public policies was mainly based on the leadership and decision-making processes rather than the content of policies themselves. Lorena (pre-service) referred to an educational forum hosted by the government, saying: “People who are invited to these forums are engineers, lawyers, and there are no teachers…. We had to say a lot like: “Hey, hello, we are here! Understand? If you are going to talk about education, include us!.” Also, it is possible that teachers felt that society focused on challenges for students to learn in an online platform, but not so much for teachers, who needed to invent different ways of reacting to the new reality. Maria (8 years of teaching experience; public school) shared her frustration, saying: “Teaching is not something seen so well in our society, which is completely absurd.”

Perhaps, a novel motivational factor from the current dataset was *collegiality among colleagues*. For instance, Sugino’s (2010) Japanese EFL teachers did not see the relationship among colleagues as an important factor, while other studies did not identify interpersonal relationships as a motivational factor for L2 teaching. According to SDT, one of the basic psychological needs to support motivation is *relatedness*, entailing a sense of belonging and connections (Ryan and Deci, 2020). The current EFL teachers expressed how important it is to establish strong interpersonal connections and a teamwork environment. For instance, Antonia (4 years of teaching experience; public school) explained: “My environment at work is good…. My colleagues had more experience than me and they gave me tips or taught me some things that I couldn’t think of because only experience could.” Relatedness as a motivational factor seems to require further attention in future TM research (see McLennan et al., 2021).

### The Role of Demotivators and Teaching Experience

A novel finding was the role that demotivators played (RQ2). Given the positive correlation between external TM and demotivators, it can be said that teachers who were externally motivated (e.g., parental and societal views) tended to feel the negative impact of demotivators more. From another perspective, those who were autonomously motivated to teach did not perceive demotivators as affecting their TM negatively. The qualitative findings suggest that teachers with more teaching experience tended to be autonomously motivated, which, in turn, helped them use demotivators as a driving force to further pursue their career.

For instance, after acknowledging several demotivators, Franco (13 years of teaching experience; private school) passionately explained: “I test my limits constantly; they say this class is hard. They say this doesn’t work. And, I go and make them work. Those are the things that motivate me. Being inside of the classroom motivates me more than it demotivates me…. I take these difficult situations and turn them into something positive, something that challenges me.” A similar sentiment was shared by Rosa (34 years of teaching experience, subsidized school) in relation to the impact of COVID-19. She declared that the pandemic had not broken her will to teach, although she was emotionally exhausted, saying: “It has been more exhausting but I really feel that I haven’t lost my motivation. I like what I do and I have fun.” The quote first shows intrinsic TM is crucial for teachers to stay motivated, especially during difficult times. Also, it shows that TM is a unique construct in that it can endure even when a person’s general well-being is under threat. It is clear that some experienced teachers managed to turn demotivators into a motivating force. Indeed, such a rather paradoxical impact of demotivators on TM explains why demotivators positively predicted TM in the model (see Figure 1). Also, the finding that COVID-19 had a minimal impact on TM (see Figure 2) suggests that EFL teachers in our study, especially those with abundant teaching experience, were resilient and knew how to shift the negative factors into a positive energy.

The interview findings also show that teaching experience should be treated differently from the comparison between pre-service and in-service teachers. Pre-service teachers have not worked at a school full-time and, hence, they may lack the knowledge to make an informed judgment as to what motivates or demotivates their TM (Lamb and Wyatt, 2019). First, the data show that pre-service teachers’ motivation was often regulated by altruistic reasons. Lorena, who was about to graduate from her teacher training program, said: “And when people say for example, “no, we have to change education” or “no, teachers don’t earn much,” it motivates me in an intrinsic way, like saying: “you know what, you’re wrong, it’s super important, we have to value the work of teachers.” Rodrigo (pre-service) commented: “That sparkle in their (children’s) eyes saying, ‘I get it. How could I miss that before?’ is what I love the most about teaching.” However, it appears that pre-service teachers, unlike experienced teachers, felt a negative impact of demotivators. Lorena also said: “Personally, projecting myself into the future, I feel that something that could as well diminish my motivation is the conditions of the context. For example, in my practicum there were 45 students.” In sum, demotivators were associated with TM differently, depending on teaching experience, and this teaching experience determined the ultimate impact of demotivators on TM.

### Learning a Second Language or Teaching It

Based on the prediction that motivation underlying TM and L2 motivation share core psychological needs, the current study examined the relationship between L2 motivation and TM (RQ3). Unlike previous research (e.g., Rahmati et al., 2019), results showed a weak relationship between L2 motivation and TM, indicating that motivation to learn an L2 and teaching the L2 are rather separate constructs. This disassociation is evident from the interview data as well. For instance, only 18% of motivators were related to *English as a language* and there were no language-related demotivators or mediators. Results offer support for Kubanyiova (2006), who characterized L2 motivation as a wrong type of TM. This does not mean that L2 motivation negatively impacts TM, however. What influenced the limited association seems to be, again, teaching experience. All 10 comments that were coded as *English as a language* were made by either pre-service or early career teachers. This pattern suggests that L2 motivation may encourage people to choose the teaching profession, but it does not necessarily help them to stay in the occupation.

Though TM and L2 motivation as two constructs were weakly associated, a closer look at the relationships among the subconstructs provides credence to SDT. The correlation results showed that those who are intrinsically driven to learn the language (e.g., future vision as an L2 user) are intrinsically motivated to teach (e.g., personal enjoyment). The flip side of the coin is that those who are motivated to teach *via* external factors (e.g., students’ parents) are those who learn the language based on external factors (e.g., societal judgment). Again, results point to the importance of autonomous motivation (cf. controlled) for learning an L2, teaching an L2, or reducing perceived burnout.

## Conclusion and Implications

With EFL teachers in Chile, the current study showed TM’s role as a deterrent of burnout. Unique to this study was the examination of the role of demotivators. The quantitative and qualitative findings suggested that teaching experience was the key for teachers to feel internally motivated and to turn the impact of demotivators into a motivational force. Similarly, experienced teachers managed to make a positive of COVID-19, and use the otherwise negative working environment it created as internal regulation, such as skill development for technology use.

Based on the findings, we recommend several future directions. First, the current study newly developed a demotivator questionnaire. Future research can examine whether the instrument is reliable in other teaching contexts. It should be noted, however, demotivators may be context specific. Second, some of the new constructs emerging from the qualitative data lend themselves to quantitative instrument development. For one, *collegiality among colleagues* may be interesting to further advance our understanding of TM from a socio-psychological perspective. Third, although the qualitative data helped understand the nuanced relationships among TM, demotivators, and burnout, the current study took a snapshot of teacher psychology that may actually be dynamically fluctuating throughout teachers’ careers (Hiver et al., 2018). Future research must investigate TM with a longitudinal design, so that we will be able to know which factors influence TM over time, and hopefully arrive at strategies to deter teacher burnout. Fourth, after knowing what demotivates L2 teachers, a logical next step is to conduct intervention studies designed to motivate teachers (see Sato and Csizér, 2021). Fifth, the current study did not examine TM’s impact on student learning (see Kubanyiova, 2019). While teachers’ well-being is important on its own, the ultimate recipients of teacher psychology are students. It is recommended that future research consider student learning as an outcome variable.

We conclude with a few practical implications. Given TM’s impact on perceived burnout, professional development workshops can include activities designed to raise teachers’ awareness of TM and demotivators. As *collegiality among colleagues* was found to be a significant mediator, schools can organize activities and events with the aim of increasing collaboration and interpersonal relationships among teachers, administrators, and parents. Such an effort may increase teachers’ well-being in general. For pre-service teachers, it is advisable that teacher education programs consider TM as a component of initial teacher training (Lamb and Wyatt, 2019). One way to integrate TM into the training curriculum may be to help pre-service teachers understand demotivators that they may face once they start to teach. It may be equally important to educate pre-service teachers on how TM is related to burnout, so that their higher-order thinking about their psychology and working environments may be stimulated. A simple yet powerful activity would be to invite experienced teachers and ask them to share their experiences. Pre-service teachers may learn not only the reality of teaching but the importance of autonomous TM to staying in the occupation. Communications with experienced teachers may also trigger *relatedness* of pre-service teachers. Finally, though it may not be possible to change societal views of the teaching job, the government certainly can reduce the gap between different school sectors in terms of their financial resources. Teachers are vital to our society. It may be important to separate *education* as a construct from *teachers* as agents who have individual psychological needs. The current study offers some ideas for recruiting teachers, helping them stay in the occupation, and taking care of their mental health.

## Data Availability Statement

The raw data supporting the conclusions of this article will be made available by the authors, without undue reservation.

## Ethics Statement

The studies involving human participants were reviewed and approved by Comité de Bioética, Facultad de Educación y Ciencias Sociales, Universidad Andrés Bello. The patients/participants provided their written informed consent to participate in this study.

## Author Contributions

MS led the project and contributed to the conceptualization of the study, research design, data analysis, and manuscript drafting. FF contributed to data collection and data analysis. JO contributed to data analysis. All authors contributed to the article and approved the submitted version.

## Conflict of Interest

The authors declare that the research was conducted in the absence of any commercial or financial relationships that could be construed as a potential conflict of interest.

## Publisher’s Note

All claims expressed in this article are solely those of the authors and do not necessarily represent those of their affiliated organizations, or those of the publisher, the editors and the reviewers. Any product that may be evaluated in this article, or claim that may be made by its manufacturer, is not guaranteed or endorsed by the publisher.
