# Supplementary material for: Teacher Motivation and Burnout of English-as-a-Foreign-Language Teachers: Do Demotivators Really Demotivate Them?
Source: Front Psychol. 2022 Apr 27;13:891452. doi: 10.3389/fpsyg.2022.891452 (PMC9094067; doi:10.3389/fpsyg.2022.891452)
Supplement: Supplementary file 1 [file Table_1.DOCX]

Supplementary Material

**Questionnaire**

**Autonomous Motivation for Teaching (α = .79)**

External Motivation

1. When I devote time to individual talks with students, I do so because I want the parents to appreciate my knowledge and familiarity with their children.

2. When I try to find interesting subjects and new ways of teaching, I do so because I want the parents to be satisfied so they won’t complain.

3. When I invest effort in my work as a teacher, I do so because I do not want the principal to follow my work too closely.

4. When I invest effort in my work as a teacher, I do so in order to prevent disruptions and discipline problems during the lessons.

Introjected Motivation

5. When I try to find interesting subjects and new ways of teaching, I do so because I think it is a shame to keep on teaching in the same way all the time.

6. When I invest effort in my work as a teacher, I do so because if I do not invest enough I would feel ashamed of myself.

7. When I invest effort in my work as a teacher, I do so because otherwise I would feel guilty.

8. When I devote time to individual talks with students, I do so because it makes me feel proud to do this.

Identified Motivation

9. When I try to find interesting subjects and new ways of teaching, I do so because it is important for me to keep up with innovations in teaching.

10. When I devote time to individual talks with students, I do so because I can learn from them what happens in the classroom.

11. When I invest effort in my work as a teacher, I do so because it is important for me to make children feel that I care about them.

12. When I invest effort in my work as a teacher, I do so because it is important for me to feel that I help people.

Intrinsic Motivation

13. When I try to find interesting subjects and new ways of teaching, I do so because it is fun to create new things.

14. When I invest effort in my work as a teacher, I do so because I enjoy finding unique solutions for various students.

15. When I invest effort in my work as a teacher, I do so because I enjoy creating connections with people.

16. When I devote time to individual talks with students, I do so because I like being in touch with children and adolescents.

**Teacher Demotivators (α = .79)**

1. Students’ attitudes during class (e.g., talking to each other, using cell-phone, forgetting to do homework, sleeping)

2. Students’ motivation to study English

3. Students’ lack of participation in activities

4. Students’ low evaluation of my teaching

5. The government’s and/or my school’s curriculum guidelines

6. Preparing students for English tests

7. High workload

8. Lack of teaching resources

9. Low pay

10. Little appreciation from my school, students’ parents, and the society

11. Please describe factors that DEMOTIVATE you to teach English. You can choose one factor and explain it or list multiple factors.

______________________________________________

**Perceived Burnout (α = .76)**

1. I feel emotionally “worn out” from teaching.

2. I feel “wiped out” at the end of a teaching day at school.

3. I feel exhausted in the morning, when I have to leave for another teaching day at school.

4. I feel that working with students for a full day is an oppressive effort.

5. I feel that teaching gives me great satisfaction.

6. I feel really “burned out” from teaching and working with students.

7. I feel that teaching frustrates me.

8. I feel that I have to work too hard in teaching.

9. I feel that teaching allows me to utilize my fullest abilities.

10. I feel that working closely with students creates a great deal of tension in me.

11. I feel really “fed up” with teaching and working with students.

12. I have considered leaving teaching.

13. I feel that teaching is turning me into an impatient person.

14. I think that I would choose to go into teaching again, if I could start my professional life over.

15. I feel that as a teacher I am not “getting ahead” in life.

**L2 Motivation (α = .81)**

Ideal L2 Self

1. I can imagine myself living abroad and having a discussion in English.

2. I can imagine myself studying in a university where all my courses are taught in English.

3. Whenever I think of my future career, I imagine myself using English.

4. I can imagine a situation where I am speaking English with foreigners.

5. I can imagine myself living abroad and using English effectively for communicating with the locals.

6. I can imagine myself speaking English as if I were a native speaker of English.

7. I imagine myself as someone who is able to speak English.

8. I can imagine myself writing English emails/letters fluently-

9. The things I want to do in the future require me to use English.

Ought-To L2 Self

10. I study English because close friends of mine think it is important.

11. Learning English is necessary because people surrounding me expect me to do so.

12. I consider learning English important because the people I respect think that I should do it.

13. If I fail to learn English, I’ll be letting other people down.

14. Studying English is important to me in order to gain the approval of my peers/teachers/family/boss.

15. I have to study English because if I do not study, I think my parents will be disappointed with me.

**Interview prompts**

1. What made you want to become a teacher?

2. What aspects of your job motivate you to teach?

3. What aspects do you think could improve your teaching motivation in the future?

4. Do you think the school environment affects your motivation?

5. Do you think your colleagues play an important role in your motivation?

6. Do you think the educational policies of your school play an important role in your motivation? What about government policies?

7. Can you explain some of the difficulties you have faced in being a teacher?

8. On a day-to-day basis, what discourages you from teaching?

9. What aspects could diminish your teaching motivation in the future?

10. Do you think your teaching motivation has changed during your teaching career? If so, what affected the changes?

11. (for pre-service teachers): What aspects do you think could improve your future teaching motivation?

12. (for pre-service teachers): Do you think your environment (people close to you) affects your motivation to become a teacher?

13. (for pre-service teachers): Do you think that the social views of teaching profession affect your teaching motivation?
